# Young peoples' involvement in welfare service development—Is voice enough?—A thematic synthesis of qualitative studies

**DOI:** 10.1111/hex.13485

**Published:** 2022-03-23

**Authors:** Line Nortvedt, Cecilie F. Olsen, Hege Sjølie

**Affiliations:** ^1^ Department of Nursing and Health Promotion, Faculty of Health Sciences OsloMet—Oslo Metropolitan University Oslo Norway; ^2^ Department of Physiotherapy OsloMet—Oslo Metropolitan University, Faculty of Health Sciences Oslo Norway; ^3^ Faculty of Health Studies VID Specialized University Theodor Dahls vei 10 Oslo 0370 Norway

**Keywords:** adolescent, patient participation, qualitative research, social participation, social welfare, systematic review, youth

## Abstract

**Background:**

Young people need to be heard and take an active role in developing welfare services. When they are recognized as having skills and expertize, the advantages young people's involvement brings to both themselves and the organizations, are mobilization and empowering with impact on national decision‐making.

**Objective:**

To synthesize existing literature on how young people's involvement in coproduction can contribute to better welfare services.

**Search Strategy:**

We performed a systematic literature search in four databases (MEDLINE, EMBASE, PsycINFO and Cinahl).

**Inclusion Criteria:**

Publications whose abstracts contained themes as: Young people 12–25 years of age, receiving welfare, youth coproduction/involvement/participation and qualitative studies.

**Data Extraction and Synthesis:**

Of the 5469 documents retrieved, the full text of 58 studies was read, of which seven studies met the inclusion criteria. A thematic synthesis following Thomas and Harden was used.

**Main Results:**

Young people being involved in coproduction of developing welfare services experienced to be valued and supported by partnerships, but they also pointed out deficiencies in welfare services. Some of the adolescents expressed not being listened to, lack of trusted relations and not being involved in policy making or prospects. The staff members saw some challenges with partnering with youth; as the need for flexibility, to keep the youth engaged and to purposefully meet the adolescents where they need help, guidance or resources.

**Conclusions:**

More involvement should be stressed. Coproduction is often symbolic more than resulting in real changes in the welfare services. Consequently, what is crucial when young people are involved is that they are encouraged by adults to be clear about the degree of involvement they want.

**Patient or Public Contribution:**

Patient and public involvement was not explicit in this review.

## 1 INTRODUCTION

Young people need to be heard and to take an active role in developing welfare services. Involvement as coproducers results in services that are more relevant, predictable and suited to young people's requirements.^1^, ^2^ When they are recognized as having skills and expertize, the advantages that young people's involvement brings to both themselves and the organizations include mobilization and empowering with an impact on national decision‐making.^3^ Including young people in decision‐making processes reduces the imbalance of power. Even so, young people today possess knowledge that is crucial for making good decisions on their own behalf.

A variety of terms are used in the literature, such as interventions, services, treatment, participants, service users, patients and consumers. We have chosen the term ‘service user’, defined as a person who utilizes health and/or social care services from service providers. ‘Service’ is defined as activities designed to promote social well‐being and/or medically necessary services, including confinement, treatments, procedures, tests or examinations.

Involvement in developing welfare services can manifest itself in different ways. A variety of studies describe how users participate in designing their own service.^4^, ^5^ Users are involved in testing technical solutions, such as apps or digital aids,^6^, ^7^ and participate in developing lifestyle and public health issues, such as sports, preventing overweight and the use of tobacco.^8^, ^9^ Some studies involved users as coresearchers.^10^, ^11^

The World Health Organization (WHO) has developed a framework for youth‐friendly health care, finding that ‘health services need to be accessible, equitable, acceptable, appropriate, comprehensive, effective and efficient’.^12^ Moreover, they state that ‘the participation of young people is needed to provide relevant, acceptable and effective services’.^12^ In 2012, the WHO also developed ten recommended standards of adolescent‐ and youth‐friendly health services, which include young people's rights, accessible health services, education and communication that promotes behaviour change that is consistent with youth‐friendly services.^13^

There is a leap from how user involvement and satisfaction in individual treatment are described to what can be understood as coproduction. Participatory action research with children and young people presents some positive consequences but also some barriers.^14^ A project aimed to integrate young people's experiences in the knowledge base of child and welfare education shows that some voices were valued while other voices were not taken into account, causing one to question whether it involved real coproduction.^15^

To the best of our knowledge, there seems to be a lack of research on how young people are involved in the actual coproduction of developing welfare services. The purpose of this paper is to synthesize existing literature on how young people's involvement in coproduction can contribute to better welfare services. We also asked, ‘What are the barriers and facilitators of coproduction in this population?’

## 2 BACKGROUND

### 2.1 Theoretical framework

Engaging young people in developing services can take on different forms. Coproduction relates to how users can individually or collectively participate in delivering their own treatments and services in collaboration with health personnel. Coproduction can be described as user design, delivery and review of services.^16^ Moreover, Fledderus et al.^17^ claim that, when service users can shape the service during their interactions with service staff, they will impact or adjust the features of the welfare service, and thereby avoid the services that are delivering poor quality.

However, people often say they are coproducing when they are not, and in this context, Hart's Ladder of participation—adapted from Arnstein^18^—differentiates between levels of involvement when engaging young people.^19^ It sets out eight levels of involvement from (1) young people are manipulated, (2) young people are decoration and (3) young people are tokenised at the bottom of the ladder. The three bottom steps indicate when young people are being used as having a voice but are having little or no impact when participating. This might be termed as being subject to different forms of adultism. The middle stages are: (4) young people assigned and informed, (5) young people consulted and informed and (6) adult‐initiated, shared decisions with young people. Young people who are participating are, to an increasing degree, informed about their role and choices. They are involved in informed decision‐making. The two top levels of the ladder are (7) young people lead and initiate action, and (8) young people and adults share decision‐making on upper layers. At these highest stages, young people take initiative and are involved with adult participants as equals.

In this regard, Dent and Pahor^16^ emphasize that policies used to develop user involvement in health care can have both disempowering and empowering consequences. They present a framework of user involvement and characterize three ideal types: voice, choice and coproduction. Choice is referred to here as the active involvement of users in making health‐related decisions. Users are transformed from being ‘consumers’ navigating a market to becoming citizens with certain rights dialogically in decision‐making processes. The user needs access to good information to make informed individual choices.

The users' involvement as coproducers results in an increase in uncertainty for Public Service Organizations, and strategies designed to minimize uncertainty as much as possible might lead to excluding certain user groups. This can also affect users' abilities to influence service performance. This lack of inclusion and influence from users can lead to reduced trust in public services and authorities.^17^

Hart's^19^ ladder provides perspectives on how young people can be invited into current shared decision‐making processes. The articles included in our review touch upon the questions of voice, choice or actual coproduction.

## 3 METHODS

A metasynthesis aims to provide a coherent overview of the literature on a chosen topic that is both faithful to the primary studies and distinct in offering a more comprehensive interpretation. Rather than merely summarizing findings, a synthesis goes above and beyond the primary study reports.^20^, ^21^ The reporting of this synthesis adheres to the enhancing transparency in reporting the synthesis of qualitative research (ENTREQ) statement.^22^

### 3.1 Search strategy

In close collaboration with a university librarian, the first author conducted a comprehensive systematic literature search using four databases (MEDLINE, EMBASE, PsycINFO and Cinahl). The publication time frame ranged from January 2010 to January 2020. With this, we aimed to achieve a balance that captured the historical legacy of youth‐friendly welfare services but focused on contemporary evidence. Grey literature and references lists were also searched.

We included the following keywords: young people, welfare services and involvement/coproduction. Suitable synonyms and subject headings were entered individually and in combination, in full spelling and truncated. We then limited the search to qualitative studies.

### 3.2 Selection criteria

We included studies undertaken in a Global North context to ensure inclusion utilizing relevant literature of appropriate mechanisms (e.g., mutuality). However, we acknowledge that findings from other health systems do not always transfer well to all European settings,^23^ and may yield indicative rather than definitive findings. Consequently, we excluded studies from low‐income countries. Furthermore, we included qualitative studies reporting on the experiences of young people or professionals in which younger people were involved in designing welfare services. The term ‘young people’ was defined as people who were 12–25 years old. Studies in which young people participated in research concerning the development of youth welfare services were included. We excluded studies on services for homeless young people and youth with a physical disability or chronic illness. We also excluded studies reporting on experiences of treatment participation, technological studies (e.g., testing apps/digital aids) and services or programmes for lifestyle or public health issues, such as obesity, healthy food, sports and tobacco‐use prevention. We included studies using a mixed‐methods design if the qualitative data were extractable from the study's results section. Studies written in languages other than English or Scandinavian were excluded due to the lack of resources for translation.

### 3.3 Screening process

**Figure 1:**
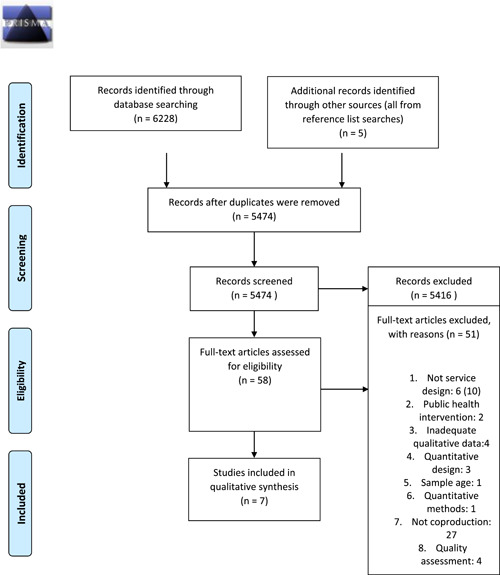
PRISMA 2009 flow diagram. PRISMA, Preferred Reporting Items for Systematic Reviews and Meta‐Analyses

We initially identified 5469 hits via the database search, zero hits in grey literature and five hits via a references list search (Figure 1). Duplicates, reviews, book chapters, poster abstracts, master's theses, commentaries and editorials were excluded. Two authors independently reviewed the titles and abstracts according to the selection criteria. Disagreement was solved by a discussion with a third author (Table 1). Finally, the full text of 58 studies was read. The authors ultimately selected 11 peer‐reviewed journal articles for a quality appraisal (Tables 2, 3, 4).

**Table 1:** Overview of the selection criteria and search elements for the metasynthesis

Criteria	Inclusion	Exclusion	Search element
Study design	– Qualitative studies	– Qualitative studies where no human subject participated (discourse analysis, textual analysis)	Qualitative studies^a^
	– Studies with a mixed‐methods design if qualitative data were extractable from the results section of the study	– Mixed‐method studies in which qualitative findings cannot be separated from quantitative findings	
Time frame	January 2010–February 2020		
Language	English and Scandinavian	All other languages	
Population	– Youth 12–25 years	– Homeless youth	Young people
	– Welfare service providers	– Youth with a physical disability or chronic illness	
Setting	– Developing countries	– Low‐income countries	Welfare services
	– Youth welfare services (health care and social care)		
Phenomenon of interest	Youth involvement in welfare service development	– Treatment participation	Youth involvement
	– Youth involvement in research aiming to develop youth welfare services	– Technological studies (e.g., testing apps/digital aids)	
	– Youth‐friendly welfare services	– Studies reporting on services or programmes for lifestyle or public health issues, such as obesity, healthy food, sports and tobacco‐use prevention	

^a^ Included as a filter in the search strategy.

**Table 2:** Quality assessment of the included studies using the JBI‐QARI appraisal instrument

Study	Canas et al. (2019)^24^	Hartas and Lindsay (2011)^25^	Heimer et al. (2018)^25^	Graham et al. (2014)^26^	Mayer and McKenzie (2017)^27^	Timor‐Slevin and Krumer‐Nevo (2016)^28^	Zlotowitz et al. (2016)^29^
Item							
Is there congruity between the stated philosophical perspective and the research methodology?	N/A	Y	Y	Y	Y	Y	Y
Is there congruity between the research methodology and the research question or objectives?	Y	Y	Y	Y	Y	Y	Y
Is there congruity between the research methodology and the methods used to collect data?	Y	Y	Y	Y	Y	Y	Y
Is there congruity between the research methodology and the representation and analysis of data?	Y	U	Y	U	Y	Y	Y
Is there congruity between the research methodology and the interpretation of results?	Y	Y	Y	U	Y	Y	Y
Is there a statement that locates the researcher culturally or theoretically?	N	N	N	N	Y	Y	Y
Is the influence of the researcher on the research, and vice versa, addressed?	N	Y	N	Y	U	Y	N
Are participants, and their voices, adequately represented?	Y	Y	Y	Y	Y	Y	Y
Is the research ethical according to current criteria or, for recent studies, is there evidence of ethical approval by an appropriate body?	U	Y	Y	Y	Y	Y	Y
Do the conclusions drawn in the research report flow from the analysis or interpretation of the data?	Y	U	Y	Y	Y	Y	Y

Abbreviations: N, no; N/A, not applicable; U, unclear; Y, yes.

**Table 3:** Overview of included primary studies

Author year	Title	Country and setting	Aim	Coproduction/participation methods	Design	Sample characteristics
					Approach and data collection methods	
Canas et al. (2019)^28^	What makes for effective, sustainable youth engagement in knowledge mobilization? A perspective for health services	Canada	To better understand the experiences and impact of youth engagement in the services	*Knowledge synthesis report*: Youth as coleads. Youth workshops to review each knowledge synthesis report before finalized	Qualitative participatory evaluation research	Five youth advisors (interviewed twice), one Wisdom2Action staff and two board members
		Youth advisory councils within the Wisdom2Action a Canada‐wide network that focuses on improving mental health services to youth in the youth service sector. No‐profit organisations				
				*Funding*: Youth cocreated fund guidelines		
				*Wisdom2Action events*: Youth designed materials, reviewed applications and helped design overall format and approach. Youth participate in planning teams and lead events as part of youth hosting team. Youth cofacilitate training and coled youth engagement	Individual interviews and textual sources from the Youth Advisory Councils	
				*Academic*: Youth lead conference submissions, cofacilitated presentations and coauthored journal publications		
Hartas and Lindsay (2011)^32^	Young people's involvement in service evaluation and decision making	UK	1. To encourage young people to offer their views about the availability and effectiveness of services with regard to bullying, disability and caring responsibilities	Young people evaluated existing services implemented at school or in their communities, and participated in decision making to recommend strategies that were deemed to be effective by the young people themselves	Qualitative approach	54 participants aged between 11 and 16:
		12 Different youth centres in geographically diverse locations in the West Midlands (England)			Focus group interviews	− 17 Young carers
						− 19 Young people who had experienced bullying
						−18 Young people with learning difficulties

			2. To explore their decision making on issues, bullying in particular, across the micro‐ and macrocontexts of their life			
Heimer et al. (2018)^32^	Vulnerable children's rights to participation, protection, and provision: The process of defining the problem in Swedish child and family welfare	Sweden	How does the children's participation in framing the problem affect the protection and provision offered to them by social services?	Children's' participation was analyzed in the written case files of 40 children (20 from each municipality). Emphasis was put on the importance of who had the right to voice in the process, analyzing different actors' competing descriptions of the problem. Three dimensions of a policy‐relevant frame were analyzed: The framings of the problem, the solutions to the problem which in turn may impact the design of care, and who is given voice to influence the framing of the problem	Mixed qualitative and quantitative	46 Social workers and family workers
		Swedish child and family welfare services in two middle‐size municipalities in different parts of Sweden				
					Case analysis	
					Written documentation of 40 child welfare investigations combined with interviews with the professionals who have carried out the investigation or provided the care	
	

Graham et al. (2014)^31^	User‐generated quality standards for youth mental health in primary care: A participatory research design using mixed methods	UK	To develop user‐generated quality standards for young people with mental health problems in primary care using a participatory research model	Young people participated in the developing and ranking of 46 youth‐friendly quality of care standards for young people with mental health problems in primary care. Coproduction followed four phases:	Qualitative Participatory research framework	− 50 Young people (aged 16–25) from community settings and primary care participated in focus groups and interviews
		The study was conducted across four South London Primary Care Trusts, the local unit for the management of primary care and community services	Feasibility of using participatory research methods in order to develop user‐generated quality standards		Focus groups and individual interviews	

				*Phase 1*: Young service users were recruited to take part in coresearcher training		A second group of young people (*n* = 12) (referred to as young service‐users) who had sought help for any mental health problem from primary care or secondary care within the last 5 years were trained as focus groups cofacilitators

		Data collection took place at sixth form colleges, a university, a drug and alcohol drop‐in center, a hostel and a research institute		*Phase 2*: Three pilot focus groups and three interviews with students and young people from a local homeless shelter and a local Muslim women's group were conducted to develop the topic guide, confirm that feedback to participants was feasible, and inform sampling and recruitment strategies		
				*Phase 3*: Developing the quality standards: participatory research groups with coresearchers		
				*Phase 4*: Developing the quality standards: reaching consensus among young service users		
Mayer and McKenzie (2017)^27^	‘…it shows that there's no limits’: The psychological impact of coproduction for experts by experience working in youth mental health	UK	To explore the what, why and how of coproduction: What is the psychological impact of coproduction on young people who are experts by experience?	Young people were paid ‘experts by experience’ for a young person's mental health charity	Qualitative phenomenological approach	Five experts by experiences, paid charity workers; mean 25 years, all male
		Mental health charity working with young people in a large ethnically diverse urban area in the United Kingdom			Individual semi‐structured interviews	
Timor‐Slevin and Krumer‐ Nevo (2016)^29^	Partnership‐based practice with young people: Relational dimensions of partnership in a therapeutic setting	Israel	To investigate partnership as experienced by people at the Youth Center	At the time of the study, around 30 young people attended the Youth Center regularly, serviced by three or four youth workers. The Center's activities were initiated by youth service users and involved a combination of structured (e.g., music, cooking and football) and open space activities. Youth workers did not compel youth to participate in structured activities, but they actively facilitate the deeper participation of youth in the Center's operations, by involving them in initiating ideas	Qualitative phenomenological approach	Several stakeholders in the youth centre, mangers, former service users (mean 23 years) and youth workers
		Partnership‐based youth center seeking to give marginalized youth a safe, nonjudgmental place to facilitate the formation of stable and close relationships with adults, including youth workers		An important medium for participation and partnership was the so‐called ‘Assembly’: A twice‐monthly meeting of everyone with a connection to the Center—managers, youth workers and youth service‐users. All are permitted to speak at these meetings about their aspirations for the Center, and mutual decisions concerning the Center—e.g., summer programs, alcohol use, budget planning	Two focus groups (*n* = 4 youth workers)	Most of the youth service users contended with socioeconomic difficulties like poverty, social marginalization and exclusion from educational settings
					10 Semi‐structured in‐depth individual interviews, participant observation of two meetings	
Zlotowitz et al. (2016)^30^	Service users as the key to service change? The development of an innovative intervention for excluded young people	UK	To explore/describe the intervention, Music & Change, an innovative complex intervention codesigned by youth to meet the needs of excluded young people	The project activities were developed in partnership with the young people, who named the project Music & Change. As led by young people, it became centred on using contemporary music skills (e.g., lyric writing and DJ‐ing) as a vehicle for building relationships and over time helping youth in ways they requested, including supporting their mental health. For example, adult practitioners (psychologists, occupational therapists and volunteers) worked with young people to facilitate a workshop on ‘making it in the music industry’ which included discussions about relationship building, confidence and cannabis	Qualitative focused ethnography	15 Young people (aged 16–22), staff and other stakeholders
					Participant observation, interviews and conversations with young people, stakeholders and staff	
		Inner‐city high‐density housing estate with approximately 500 apartments. The area fell within the 14% most deprived similar‐sized areas in the United Kingdom				

**Table 4:** Example of the thematic analysis^30^

Examples of codes	Descriptive themes	Analytical themes
Being valued	Youths' experiences of being involved	Mutuality of gain
Partnership		
Engagement		
Support		
Belonging and connectedness		
Changed identity		
Being included		
Keeping youth engaged over time	The advantages and challenges with youths' involvement	Challenges of partnering with youth
Need for youth‐friendly skills		
Support yes, interference no		
Avoiding adultism		
Being heard	The process of involving youth	Voices that ebb out
Not being heard		
Varying degrees of choice		
Limited follow‐up by the organization		

### 3.4 Quality appraisal

Two authors independently conducted a quality appraisal of 11 articles using the Critical Appraisal Skills Programme, JABRI.^31^ The authors discussed any ambiguities regarding the quality of the studies until reaching an agreement. Finally, we excluded four articles based on low quality (defined as less than 6 out of 10 Yeses on the checklist), leaving seven articles for the final thematic synthesis.

### 3.5 Characteristics of the primary studies

This metasynthesis is based on findings from seven primary studies undertaken in the United Kingdom (4), Israel, Canada and Sweden between 2011 and 2019. An overview of the studies is provided in Table 1. The studies included experiences from over 60 different types of service providers and over 100 young people involved in youth welfare service development. The study samples ranged from 5 to approximately 50.

The studies were heterogeneous and varied considerably with regard to methodological approach, setting and sample size.

### 3.6 Analysis and synthesis

Thematic synthesis following Thomas and Harden^30^ was used. This analysis has three stages: line‐by‐line coding of the text, development of descriptive themes and generation of analytical themes. While the development of descriptive themes remains ‘close’ to the primary studies, the analytical themes represent a stage of interpretation whereby the reviewers generate new interpretive constructs or explanations. As Thomas and Harden^30^ recommend, the results sections of the included articles were coded and analysed using the computer software NVivo12.^32^ The results section from all seven of the evaluated studies were extracted and copied into a single Word document, which was uploaded to NVvivo. Two authors (L. N. and H. S.) coded the extracted data separately, and developed descriptive themes, primarily inductively. All of the authors were involved in the final development of the analytical themes; at this stage, we applied relevant theory. It was an advantage that the authors consisted of a nurse (L. N.), a physiotherapist (C. O.) and a sociologist (H. S.), as the findings could be analysed and assessed from different points of view. The studies were rather heterogeneous and represented a challenge in the analysis; however, the concepts from the theoretical framework proved useful for facilitating the analysis across studies.

## 4 FINDINGS

With this metasynthesis, our overall aim was to synthesize existing literature on how young people's involvement in coproduction can contribute to enhanced welfare services. The main finding that emerged from our analysis of the studies was: ‘Is voice enough when young people are involved in welfare service development?’ The results covered three broad themes: (1) Mutuality of gain, (2) The need for adaptation when partnering with young people and (3) Voices that fade away. All names are anonymized.

### 4.1 Mutuality of gain

The analysis showed that both adults and young people benefited from being involved in developing welfare services. The young people who became involved in such collaboration reported feelings of being valued and supported.^24^, ^27^ Participation resulted in the young people stating that the network they were engaged in was useful and they specifically mentioned supportive mentorship. One youth said, ‘I feel totally engaged with the Network, and quite valued… I feel supported as a youth, and that my agenda is supported’.^24^

Moreover, the young people noted that they felt that someone was concerned about them and that they received support, reassurance and respect from staff. The young people felt that they could impact others and they were valued for their experience and knowledge.

In addition to mentioning the positive experiences of being included in coproduction and networking, the young people gained a sense of equality with staff members,^24^, ^27^ as well as a sense of belonging and connectedness.^28^, ^29^ For some of the young people, involvement meant developing professional identities and leadership skills.^24^, ^27^, ^28^ One of the youths in Mayer and McKenzie's^27^ study stated,

*I'm a professional, yeah, we're both professionals, both on the same level. But level, they're, obviously, there, like he's a supervisor, I'm the youth engagement practitioner, but, still professionals. Both of us, as simple as that*.

According to Zlotowitz et al.,^29^ the young people sought support for stability, jobs and socioeconomic improvements over a longer period, and these matters were more vital to them than emotional support. Further, they wanted resources, opportunities and allies so that they could escape perilous environments and find safer ones.

Furthermore, many of the young people experienced trusted relationships and supportive partner‐ and mentorships with adults.^27^, ^28^, ^29^ In contrast, in Hartas and Lindsay's^25^ study, the young people missed having trusting mentorships.

These findings demonstrate the importance of mutuality of gain as the staff members or adults working with young people also experienced gains and advantages when adolescents were involved in the coproduction of welfare services. The collaboration contributed to increased creativity in the services and clearer ways of prioritizing activities and plans. Feedback from the young people on different types of work led to improvements in the programme and enhanced their well‐being.^24^ The mentioned mutuality is demonstrated in the following quote from a youth worker:

*I believe it's a process we go through together, over time; crises, happy events, it's something that binds, it isn't one specific thing. What's meaningful is the moment when each of the parties influences the other. It was something very, very mutual, very, very strong, a real connection…*. (1YW—Naama, youth worker)^28^

Despite mutual gains for the youth and adults, designated through our analysis, we also found that the staff experienced a need for adaptation.

### 4.2 The need for adaptation when partnering with young people

A central challenge in partnering with young people seemed to be balancing the degree of support and independence given to young people when developing and maintaining services. Some of the studies highlighted the need for different skillsets and skills building in the adult group.^24^, ^26^ The adults identified a need for more training in participatory approaches, specifically regarding the language used, how to perform mentorship and how to contribute to social enterprise models.^24^ In the study by Canas et al.,^24^ the adults also pointed to the challenges of ensuring better documentation and tracking of processes for the youth councils.

The young people emphasized the need for safe and accessible physical and social spaces, such as safe parks, where they could meet with friends and foster relationships. They also emphasized the need for support from adults and parents;^25^ however, it appeared crucial that adults did not define the problem or structure the activities because the young people viewed this as having too much control over the environment. One youth said that there was ‘too much safety at the school and some of the things are ridiculous’, commenting that an ‘overly structured and adult‐supervised space’ poses restrictions on their enjoyment of activities or trips.^25^ Here, this is interpreted as the need for adaptation by seeing the problem from the young people's point of view and letting them define the problem.

A recurrent theme in some of the studies is the young people's relationships with their parents. The social workers and family workers in Heimer et al.'s^33^ study described the importance of avoiding blaming the parents and teaming up with them, so that the parents agreed to receive support, for example, in setting boundaries and having a structure in the family life. One participant said, ‘…we always work with what the parents wish to get help with’.^33^

The challenges of partnering with youth seemed related to diversity, equality and asymmetric power relations. A leader in the study by Timor‐Shlevin and Krumer‐Nevo^28^ described it like this: ‘…we talk about repair from within a system of domination, of one dominating the other, so that partnership is itself the reparation’.

Here, partnership is a process of adaptation through which the participants recognize, work through and find their footing in unequal relationships. Asymmetric power relations are the motives for establishing a partnership as it helps repair the negative impact of domination, submission and ferocity that can occur in situations where there is an unequal power balance.

Another lesson learned was the need for flexibility and adaptation to young people's changing circumstances. The adults found it was challenging to keep young people engaged over time, and they were confronted with the dilemma of rewarding them in terms of paid or unpaid work.^24^

A decisive factor for ensuring the young people's engagement seemed to be the need to take account of the shifting conditions of their everyday lives, as well as variations in their well‐being.

Moreover, Timor Slevin and Krumer‐Nevo^28^ pointed out that young people's coproduction can be seen as a holistic experience, where partnership is described in two interconnected spheres—the structural–technical and the content‐experiential—and both must be in place to hear voices. Thus partnership is an experience of self and the other, rather than a pragmatic tool for decision‐making, and it might have valuable therapeutic effects.^28^ The therapeutic effect is promoted from a warm and welcoming environment that introduces feelings of belonging, connectedness and togetherness.

Here, partnership is labelled as an ongoing, adjusting practice that presupposes an understanding of the circumstances for the partnership in order for its further development, and upon which decision‐making rests. This is contradictory in relation to our next finding, which deals with voices that are not heard.

### 4.3 Voices that fade away

Even if the participants identified many gains of coproduction, our findings also showed welfare services that were deficient. Overall, the young people felt that their voices were heard, but they also experienced that practical consequences or long‐term structural changes were lacking. However, some young people also experienced not being listened to, or that their inputs were misunderstood.^25^ Heimer et al.^33^ claimed that, when children are not given a voice to impact the framing of the problem, the strategy of protection and care tends to be poorly harmonized. Similarly, the children were seldom invited to participate in decisive meetings for family treatment, thus their opinions were not heard. One said, ‘I say things, I come with an idea, but they do not listen. Teachers have an idea and do not listen to anybody else…’.^25^

Without listening to young people's voices, the interventions from the staff might be misplaced. One youth stated,

*Instead of having too many literacy lessons just have a bullying lesson, and ask children to write down their thoughts about how bullies think; it is only in assembly that we talk about bullying but they do not stop bullying…*.^25^

When young peoples' voices were heard, a persistent challenge was whether what they said was taken into consideration at the organizational level. These can be termed as voices that fade away. In Mayer and McKenzie's^27^ study, the participants described the lack of organizational support in that decisions were made without their input, information was withheld, and they experienced a loss of power and the feeling of being a ‘lab rat’.

The youth participants in Canas et al.'s^24^ study also pointed to the lack of involvement when it came to policymaking or the future vision of the organization, as well as not receiving enough training in specific areas. Likewise, Zlotowitz et al.^29^ pointed out the need for organizational support and creating contextual changes as the young people perceived that, even though they changed, the organization or their local environment did not change, leading to a feeling of despair or decreased motivation.

In different ways, many of the studies emphasized the importance of young people contributing to defining the problem. However, even if they do, their contributions are not followed up in the organizations. As one of the youth advisors noted,

*Racialized youth are on the YAC, and we're doing the on‐the‐ground work with the youth sector, but are not necessarily reflected in the decision‐making or future‐setting of the organisation … The youth don't only need to be on the YAC, they could be involved at other levels of the organization*.^24^

The young people in Hartas and Lindsay's^25^ study provided interesting opinions on opportunities for decision‐making and possibilities to convert policy and practice across different contexts. However, they voiced concerns about decreased possibilities that their suggestions would be implemented at their schools. Consequently, voice alone was not enough when young people were involved in welfare service development, and there was a need for appropriate follow‐up at the organizational level.

## 5 DISCUSSION

The results reported in the reviewed studies showed that the young people involved in coproduction of developing welfare services felt valued, reassured, respected and supported by mentorships and partnerships. The young people also felt mutuality and equality with adults and developed professional identities. However, they pointed out deficiencies in welfare services and some described not being listened to or having a lack of trusted relations. Furthermore, the staff members saw the need for adapting when partnering with young people, such as the need to keep the young people engaged, the need for skills development, involving parents at appropriate stages, and purposefully meeting the adolescents' needs for help. In general, to a large extent, the young people in the reviewed studies felt their voices were heard. However, they also experienced a lack of involvement in framing the problems, that what they said was not considered at the organizational level, and that policy‐making or changes in the local environment were not followed up.

Our study aimed to synthesize existing literature on how young people's involvement in coproduction can contribute to better welfare services, and to identify which barriers to and facilitators of coproduction exist in this population. However, we only included seven studies, which is small; thus, there is a need and potential for more research on this topic. According to a Norwegian report about coproduction in public health work, the interest and use of coproduction have increased significantly over the course of only a few years.^34^ Moreover, two systematic review studies showed that the aim of coproduction processes varies across different countries and settings, is sporadically described and fails to focus on the processes' effects.^35^, ^36^

Regarding the degree of youth involvement in the selected papers, considering Hart's Ladder of participation,^19^ we found that, at most, the young people might be placed at rung six of the ladder because adults initiated the projects by sharing decisions with the adolescents, which is exemplified in our finding of ‘voices that fade away’. The young people were mostly consulted and informed (rung 5), and to a lesser extent, the projects were led or initiated by the young people. While Zlotowitz et al.^29^ claimed that the project was ‘led by young people … helping the young people in ways they requested’, the study was built on an intervention for excluded young people who were recruited through informal and participatory methods.

Using Dent and Pahor's^16^ framework, we also saw that young people's experiences discussed in these studies can be associated with ‘coproduction’, as they felt they had impact, mutuality and equality, and control from being experts or professionals; they also experienced partnerships and mentorships. According to Dent and Pahor,^16^ this could be described as engaging in the delivery of their services. When the adults in our reviewed studies were supportive, creative, prioritized activities and saw the need for flexibility, this could also be defined as ‘user co‐delivery of professionally designed services’.^16^ Even when the young people participated in developing user‐generated quality standards, arguing for rankings,^26^ we might equate it with coproduction.^16^ The study about bullying^25^ and the study about children impacting the framing of the problem^33^ might be termed as ‘voice’ since, to varying degrees, the children were involved in decision‐making.^16^

Regarding barriers for coproduction, the adults in some of the studies^24^, ^25^ noted that they needed more economic resources to satisfactorily include the young people in the decision‐making process. This is in line with Fledderus et al.,^17^ who claimed that financial support is one of the uncertainties with which organizations must deal.^17^ Moreover, Fledderus et al.^17^ mentioned the lack of motivation to contribute as a barrier, which in our findings, is denoted as lack of engagement or the challenge of keeping the young people engaged for a longer period of time.^24^ In this regard, Fledderus et al.^17^ pointed to financial or nonmaterial rewards as the joy or intrinsic motivation of coproducing, which can be reflected in social recognition or group identity, as was the case for many of the young people in the studies we reviewed. Furthermore, the results of this study coincide with the findings from the literature on coproduction for the adult population.^36^, ^37^, ^38^

Our findings demonstrate the importance of long‐lasting relationships between youth and adults for establishing trust. However, Fledderus et al.^17^ determined this was a conceivable obstacle, as trust within a group is a prerequisite for cooperation and, thus, for successful collective coproduction. This is also recommended in the WHO^13^ standards of adolescent‐ and youth‐friendly health services, which states that youth‐friendly healthcare providers should be ‘non‐judgmental and considerate, easy to relate to and trustworthy’. Even if some of the adolescents in the studies described the lack of trusted relationships,^25^ most of the studies accentuated trusting relationships between young people and adults.^27^, ^28^, ^29^ According to two of the studies, the adult group demanded skillsets and skills building.^24^, ^26^ This is in line with the WHO^13^ standards maintaining that a youth‐friendly approach should involve continuous updating as well as the development of new skills among staff.

### 5.1 Strengths and limitations of the study

There are diverse understandings in the field of participation, and our search was complicated by the fact that there are many different definitions of the concept of coproduction (participatory action research, knowledge construction, young people's participation, codesign and collaboration).

One of the study's strengths is the use of a well‐known method, the thematic synthesis of qualitative research in systematic reviews, by Thomas and Harden.^30^ Moreover, we emphasized rigour in selecting the studies, and three authors were involved in the entire process/analysis. The selection of studies and the quality appraisal were done independently following the rules of systematic literature reviews.^39^

The analysis was based on primary studies involving different qualitative approaches in different contexts. This heterogeneity was anticipated, but it can be challenging when interpreting data from different countries, settings, service providers and youth groups. However, we see heterogeneity as a possible source of insight. Despite their differences, the primary studies were similar in reporting young people's involvement in coproduction and how this could potentially contribute to better welfare services, which gave credibility to the overall themes.

One of the limitations is that studies written in languages other than English or Scandinavian were excluded. Moreover, the relatively low number of included studies might be seen as a limitation. However, given the richness of the information obtained from each study, a larger sample could have prevented a deeper analysis of the topics, which could have threatened the interpretive validity of the results.

This paper is part of a larger project, and young people participated in framing the review study's purpose. We are, however, aware of the limitation of the study as young people did not participate in all phases of the thematic synthesis.

## 6 CONCLUSION

We conclude that, although welfare services facilitate coproduction, it turns out that this is sometimes done to satisfy the granting authorities, so that coproduction becomes symbolic rather than resulting in real changes in welfare services. Simultaneously, we see that some degree of user involvement is better than none. Consequently, what is crucial when young people are involved is that they are encouraged by adults to be clear about the degree of involvement they want. This study has important implications for future practice and research. To professionals, it implies that they need to operate in a more flexible way. To young people, coproducing services means embarking on a reciprocal relationship with professionals, other youth and relevant adults that will help them recover and learn. Future research should support practitioners to identify the appropriate conditions and practical skills, which enable coproduction to flourish. Forthcoming studies could also develop relevant tools needed to generate further evidence of the value of coproduction in youth services.

## CONFLICTS OF INTEREST

The authors declare no conflicts of interest.

## Data Availability

Data sharing is not applicable to this article as no new data were created or analyzed in this study.
